# Effect of dapagliflozin on the initial estimated glomerular filtration rate dip in chronic kidney disease patients without diabetes mellitus

**DOI:** 10.1007/s10157-022-02277-y

**Published:** 2022-09-17

**Authors:** Ryo Shibata, Kensei Taguchi, Yusuke Kaida, Kei Fukami

**Affiliations:** Division of Nephrology, Department of Medicine, Kurume University School of Medicine, 67 Asahi-Machi, Kurume City, Fukuoka, Japan

**Keywords:** Chronic kidney disease, Dapagliflozin, Estimated glomerular filtration rate, Hemoglobin, Initial dip, SGLT2 inhibitor

## Abstract

**Background:**

Dapagliflozin (DAPA), a sodium-glucose transporter 2 inhibitor (SGLT2i), attenuates kidney outcomes in patients with not only diabetes mellitus (DM) but also chronic kidney disease (CKD). SGLT2i-derived initial dip in estimated glomerular filtration rate (eGFR) has been considered to reduce excess glomerular pressure, followed by renal protection in patients with DM. However, whether DAPA confers the eGFR dip and its independent determinants for CKD patients without DM are unclear.

**Methods:**

A total of 126 patients with CKD treated with 10 mg DAPA daily was retrospectively registered. After participants with missing data and DM were excluded, 51 participants were enrolled.

**Results:**

An initial eGFR dip was observed 1 month after initiation of DAPA, which was sustained until 2 months. DAPA did not affect urinary protein excretion; however, serum uric acid was decreased, while hemoglobin level was increased. Multiple regression analysis revealed that eGFR at baseline was the only independent determinant of the initial dip of eGFR. The patients currently showing exacerbation of glomerular hyperfiltration exhibited the larger initial eGFR dip rather than those showing progressive renal dysfunction. The patients meeting exclusion criteria of DAPA-CKD trial exhibited same degree of the initial eGFR dip as others.

**Conclusions:**

DAPA causes an initial dip of eGFR in CKD patients without DM at 1 month after starting DAPA treatment. A higher eGFR at baseline predicts a large initial eGFR dip, which might be linked to the subsequent recovery in eGFR in CKD patients without DM.

## Introduction

Chronic kidney disease (CKD) is a substantial public health burden worldwide that accelerates cardiovascular disease and leads to high rates of morbidity and mortality [1, 2]. It has been demonstrated that the number of CKD patients has continuously increased in the past 3 decades and currently more than one of seven adults (15% of adults in the United States) suffer from CKD　[3]. Renin–angiotensin system (RAS) inhibitors and/or immunosuppressant therapies have been commonly used to prevent progression of kidney injury in CKD patients [4]. Despite the current development of therapeutic agents, the number of patients requiring renal-replacement therapies is expected to increase [5].

There is now accumulating evidence, showing that a sodium-glucose cotransporter-2 inhibitor (SGLT2i) has distinct beneficial effects on renal and cardiovascular outcomes of patients with type 2 diabetes mellitus (DM) [6, 7]. The EMPA-REG outcome trial has clearly demonstrated that empagliflozin reduced the risk of worsening kidney injury in patients with type 2 DM (progression to macroalbuminuria, doubling of the serum creatinine level, initiation of renal-replacement therapy, or death from renal disease) [8]. More recently, dapagliflozin (DAPA) robustly mitigated renal hard endpoints compared to placebo even in CKD patients without DM in the DAPA-CKD trial [9]. Based on effectiveness and safety of DAPA shown in the several clinical trials, DAPA has been approved for use in CKD patients in Japan.

SGLT2i has been shown to attenuate glomerular hyperfiltration via tubuloglomerular feedback system, which is linked to inhibit progression of CKD [10]. The phenomenon can be observed as the decrease in initial dip of estimated glomerular filtration rate (eGFR) instantly after starting DAPA treatment in patients with type 1 DM [11]. However, whether DAPA treatment causes the initial eGFR dip and what clinical characteristics are independent determinants for the initial eGFR dip in CKD patients without DM have not been elucidated. Thus, we have investigated retrospectively what kind of clinical parameters influence the occurrence of initial eGFR dip in the present study.

## Materials and methods

### Enrollment of DAPA-treated CKD patients without DM

**Fig. 1:**
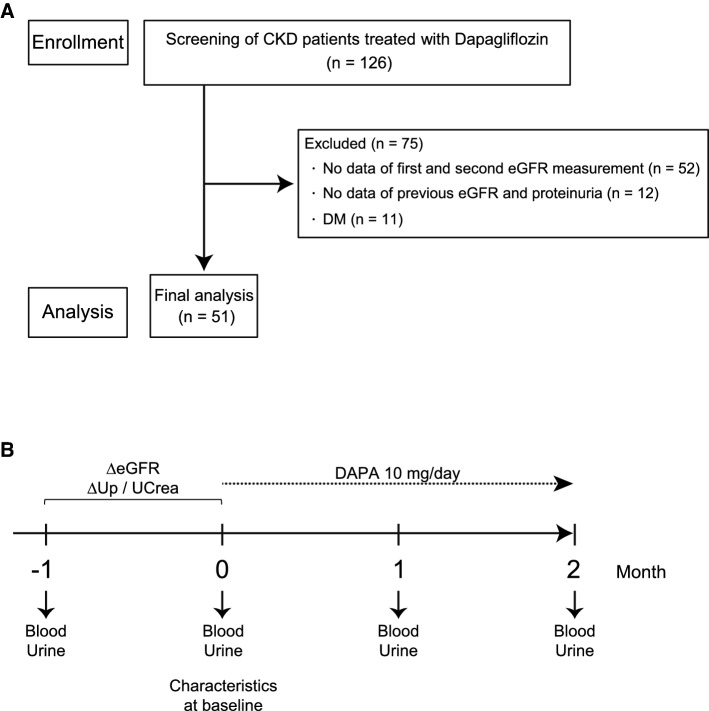
(**A**) Flowchart of the screening of CKD patients without DM treated with dapagliflozin. (**B**) Scheme of the present study with the enrolled patients. *CKD* chronic kidney disease, *DM* diabetes mellitus, *eGFR* estimated glomerular filtration fate

A total of 126 patients treated with DAPA 10 mg daily between September 2021 and March 2022 was registered. Fifty-two participants did not have eGFR data before the DAPA initiation and 12 participants did not have four-point proteinuria data (1 month and just before the DAPA initiation and 1 month and 2 months after the DAPA initiation). Based on the medical record of Kurume University hospital, 11 patients were diagnosed of diabetes or treated with anti-diabetes agents. Thus, a total of 75 patients was excluded from the present study, and the remaining 51 CKD patients without DM were enrolled (Fig. 1A, B).

### Data collection and study procedure

Data on the clinical characteristics of the participants, including age, sex, and laboratory data such as proteinuria and eGFR; cause of kidney disease; and medication use were obtained from the electronic medical records of Kurume University Hospital. eGFR was calculated using a previously described formula [12]. Changes in eGFR, urinary protein excretion levels, uric acid levels, and hemoglobin levels were evaluated before and after DAPA treatment. Independent determinants of the initial eGFR dip were investigated. In addition, patients were categorized by current glomerular filtration and proteinuria, CKD stage, and criteria of DAPA-CKD trial, and the initial eGFR dip was analyzed.

### Statistical analysis

The results are presented as the mean ± standard deviation. To compare the initial eGFR dip, urinary protein levels, uric acid levels, and hemoglobin levels before and after treatment, paired Student’s *t* test was performed. Difference in the initial eGFR dip according to CKD stage was analyzed by one-way ANOVA followed by Tukey’s post hoc test. Difference in the initial eGFR dip according to current status of glomerular filtration and proteinuria were analyzed using Dunnett’s test. Univariate and multiple regression analyses were performed to assess the independent determinants of the initial eGFR dip. All statistical analyses were performed using JMP Pro version 15 software (SAS Institute, Inc.). Statistical significance was set at *p* < 0.05.

## Results

### Baseline clinical characteristics of CKD patients without DM

The baseline clinical characteristics of all patients are shown in Table 1. The mean age was 58.1 ± 14.0 years. The mean systolic and diastolic blood pressure were 128 ± 1.6 and 62 ± 1.6 mmHg, respectively. The mean hemoglobin level, eGFR, urinary protein level, and uric acid level were 12.3 ± 1.6 g/dL, 28.7 ± 14.8 mL/min/1.73 m^2^, 2.03 ± 2.61 g/gCr, and 6.11 ± 1.14 mg/dL, respectively. The causes of kidney disease were chronic glomerulonephritis, immunoglobulin A (IgA) vasculitis, autosomal-dominant polycystic kidney disease, hypertensive nephrosclerosis, lupus nephritis, Fabry nephropathy, and unknown (Table 1). Eighty-six percent of participants were prescribed RAS inhibitors and 69% and 25% of those were prescribed calcium channel blockers and diuretics, respectively (Table 1).

**Table 1:** Clinical characteristics of the patients

No. of patients	51
Age (years)	58.1 ± 14.0
No. of male patients (%)	25 (49)
Systolic BP	128 ± 1.6
Diastolic BP	62 ± 1.6
Hemoglobin (g/dL)	12.3 ± 1.6
Total protein (g/dL)	6.91 ± 0.53
Serum albumin (g/dL)	3.91 ± 0.47
BUN (mg/dL)	32.2 ± 13.4
Serum Cr (mg/dL)	2.23 ± 1.23
eGFR (mL/min/1.73m^2^)	28.7 ± 14.8
UP/UCrea (g/gCr)	2.03 ± 2.61
Uric acid (mg/dL)	6.11 ± 1.14
LDL-cholesterol (mg/dL)	111 ± 33
Cause of kidney disease (%)	
Chronic glomerulonephritis	26 (51)
IgA nephropathy	10
FSGS	8
MN	3
MPGN	1
Unknown	4
IgA vasculitis	2 (4)
ADPKD	7 (14)
HN	9 (17)
Lupus nephritis	1 (2)
Fabry nephropathy	1 (2)
Unknown	5 (10)
Medications (%)	
RAS inhibitors	44 (86)
Ca blockers	35 (69)
Diuretics	13 (25)
Statins	20 (39)
ESA	3 (6)
HIF-PH inhibitor	5 (10)

Values are shown as mean ± standard deviation

*No.* number, *BP* blood pressure, *BUN* blood urea nitrogen, *Cr* creatinine, *eGFR* estimated glomerular filtration rate, *UP/UCrea* urinary protein/urinary creatinine ratio, *LDL* low-density lipoprotein, *FSGS* focal segmental glomerulosclerosis, *MN* membranous nephropathy, *MPGN* membranoproliferative glomerulonephritis, *ADPKD* acquired dominant polycystic kidney disease, *HN* hypertensive nephropathy, *RAS* renin–angiotensin system, *Ca* calcium, *ESA* Erythropoiesis-stimulating agents, *HIF-PH* Hypoxia Inducible Factor Prolyl Hydoxylase

### Efficacy of DAPA on eGFR and clinical parameters

**Fig. 2:**
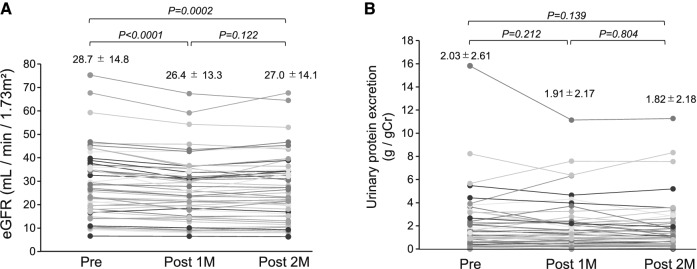
Changes in eGFR and urinary protein levels before and after dapagliflozin treatment. (**A**) Changes in eGFR and (**B**) urinary protein levels before and after the initiation of DAPA. *CKD* chronic kidney disease, *DAPA* dapagliflozin, *eGFR* estimated glomerular filtration rate

**Fig. 3:**
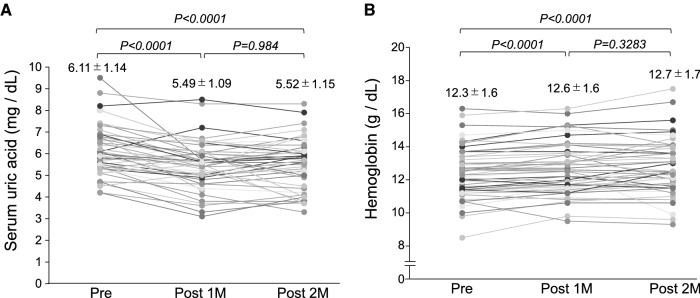
Changes in serum uric acid and hemoglobin levels before and after dapagliflozin treatment. (**A**) Changes in serum uric acid and (**B**) hemoglobin levels after the initiation of DAPA. *DAPA* dapagliflozin

eGFR was significantly decreased at 1 month and 2 months after initiation of DAPA treatment (Fig. 2A). However, urinary protein level was not changed (Fig. 2B). Serum uric acid levels were significantly decreased at 1 month and 2 months after starting DAPA treatment (Fig. 3A). Hemoglobin level was statistically increased at 1 month and 2 months after DAPA treatment (Fig. 3B). There was no change in the above parameters between 1 and 2 months (Figs. 2A, B, 3A, B)

### Determinants of the initial eGFR dip at 1 month after DAPA treatment

**Fig. 4:**
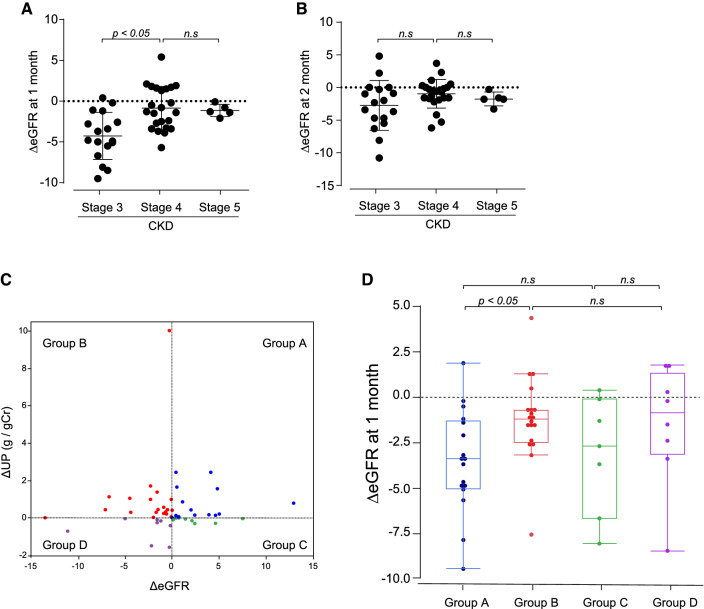
Changes in eGFR according to CKD staging and the current status of ∆eGFR and ∆urinary protein levels before DAPA initiation. (**A**) The change in eGFR at 1 month and (**B**) the change in eGFR at 2 month after the initiation of DAPA. (**C**) Quadrant chart for classifying the patients by the current status of ∆eGFR and ∆UP before DAPA initiation. (**D**) The change in eGFR at 1 month after initiation of DAPA in each group. *DAPA* dapagliflozin, *eGFR* estimated glomerular filtration rate, *UP* urinary protein

To determine the correlation between initial eGFR dip and clinical parameters, univariate and multiple regression analyses were performed. Age (positive; *p* = 0.025), hemoglobin (inverse; *p* = 0.005), blood urea nitrogen (positive; *p* = 0.002), serum creatinine (positive; *p* = 0.006), and baseline eGFR (inverse; *p* < 0.001) were correlated with the initial eGFR dip (Table 2). Furthermore, multivariate regression analysis demonstrated that baseline eGFR was an independent determinant for DAPA-derived initial eGFR dip in CKD patients without DM (*β* =  − 0.614; *p* = 0.002) (Table 2). Patients having higher eGFR seem likely to develop large eGFR dip after starting DAPA. Next, to determine if CKD stage is associated with the initial eGFR dip, the enrolled patients were classified by CKD stages and the change in eGFR at 1 month and 2 months after initiation of DAPA treatment was analyzed (CKD stage 2 was excluded because of the small number: *n* = 2). There was no significant difference in clinical parameters at baseline, including age, sex, anemia, and blood pressure among the groups (Table 3). LDL-cholesterol was statistically lower in CKD stage 5 than that in stage 3. We identified that patients with CKD stage 3 showed the larger eGFR dip at 1 month than that of patients with CKD stages 4 and 5 (Fig. 4A). However, the difference in initial eGFR dip seen at 1 month disappeared at 2 months (Fig. 4B).

**Table 2:** Univariate and multiple regression analyses for the determinants of initial eGFR dip in non-DM CKD patients

Variables	Univariate regression			Multiple regression		
	SE	*β*	*p*	SE	*β*	*p*
Age	.649	.313	0.025*			
Sex	.025	.069	0.631			
Hemoglobin	.071	− .390	0.005**	.256	− .105	0.445
Total protein	.029	− .075	0.639			
Serum albumin	.023	− .201	0.165			
LDL-cholesterol	1.615	− .285	0.064			
BUN	.592	.425	0.002**	.040	− .072	0.696
Serum Cr	.055	.382	0.006**			
eGFR	.586	− .585	< 0.001***	.038	− .614	0.002**
UP/UCrea	.128	.056	0.701			
Uric acid	.128	− .016	0.914			

Values are shown as mean ± standard deviation

*R*^*2*^ 0.400, *eGFR* estimated glomerular filtration rate, *DM* diabetes mellitus, *CKD* chronic kidney disease, *BUN* blood urea nitrogen, *Cr* creatinine, *UP/UCrea* urinary protein/urinary creatinine ratio, *LDL* low-density lipoprotein. *p < 0.05, **p < 0.01, ***p < 0.001

**Table 3:** Differential characteristics according CKD stage

CKD stage	Stage 3	Stage 4	Stage 5
No. of patients	18	20	11
Age (years)	54.3 ± 12.2	60.7 ± 13.8	62.3 ± 16.1
Sex (male)	10 (56)	9 (45)	6 (55)
Systolic BP (mmHg)	130.1 ± 14.2	129.6 ± 13.0	131.4 ± 16.4
Diastolic BP (mmHg)	81.3 ± 12.6	79.2 ± 10.1	80.8 ± 7.46
Hemoglobin (g/dL)	13.3 ± 1.53	11.84 ± 1.44	11.8 ± 1.4
Total protein (g/dL)	6.99 ± 0.45	6.80 ± 0.59	6.93 ± 0.54
Serum albumin (g/dL)	3.98 ± 0.36	3.77 ± 0.55	4.00 ± 0.44
LDL-cholesterol (mg/dL)	117.8 ± 23.7	113.4 ± 34.5	81.3 ± 25.7*
BUN (mg/dL)	23.4 ± 4.00	32.8 ± 9.40**	49.3 ± 12.7***^###^
Serum Cr (mg/dL)	1.37 ± 0.27	2.10 ± 0.33**	4.19 ± 1.04***^###^
eGFR (mL/min/1.73m^2^)	39.9 ± 7.02	23.7 ± 4.60***	11.6 ± 3.01***^###^
UP/UCrea (g/gCr)	1.63 ± 1.56	2.65 ± 3.72	1.74 ± 1.26
Uric acid (mg/dL)	6.18 ± 0.27	5.96 ± 0.26	6.09 ± 0.34

Values are shown as mean ± standard deviation or range

*BP* blood pressure, *eGFR* estimated glomerular filtration rate, *No.* number, *BUN* blood urea nitrogen, *Cr* creatinine, *UP/UCrea* urinary protein/urinary creatinine ratio, *LDL* low-density lipoprotein. *p < 0.05 vs Stage 3, ***p < 0.001 vs Stage 3, ^#^*p* < 0.05 vs Stage 4, ^###^*p* < 0.001 vs Stage 4

### Differences in the initial eGFR dip according to the current change in eGFR and urinary protein prior to the initiation of DAPA treatment

Short-term changes in intraglomerular pressure and proteinuria are known to reflect current status of glomerular filtration, which may predict the degree of eGFR dip after administration of SGLT2i. To investigate whether the current status of glomerular filtration and proteinuria is associated with DAPA-induced eGFR dip, the change in eGFR (∆eGFR) and urinary protein level (∆UP) between two consecutive laboratory test and urinalyses before initiation of DAPA was evaluated (Fig. 1B). Then, the patients were classified into four groups as follows; group A, increased eGFR plus increased UP (hyperfiltration group); group B, decreased eGFR with increased UP (glomerular injury group); group C, increased eGFR with declined UP (non-progressive group); group D, decreased eGFR with declined UP (glomerular collapse group) (Fig. 4C). There is no statistical difference in age, sex, hemoglobin, total protein, serum albumin, LDL-cholesterol, eGFR, serum uric acid, and UP/UCr among all groups at baseline. BUN is higher in group B than group A at baseline (Table 4). Nevertheless, we identified that the initial eGFR dip at 1 month was significantly larger in group A when compared to that in group B (Fig. 4D). Group A can be considered those showing current exacerbation of glomerular hyperfiltration and group B can be defined as those showing progressive renal dysfunction. Thus, this finding suggests that the patients with the sign of exacerbation of glomerular hyperfiltration are likely to show the large initial eGFR dip after initiation of DAPA treatment.

**Table 4:** Differential characteristics according to the changes of eGFR and proteinuria levels in CKD patients

Group	A	B	C	D
Current status	eGFR↑/UP↑	eGFR↓/UP↑	eGFR↑/UP↓	eGFR↓/UP↓
No. of patients	17	19	7	8
Age (years)	54.2 ± 13.6	46.3 ± 21.0	56.4 ± 8.9	53.5 ± 8.1
Sex (male)	8 (47)	13 (68)	1 (14)	3 (38)
Systolic BP (mmHg)	126.8 ± 3.55	133.0 ± 3.45	124.8 ± 5.53	134.8 ± 5.18
Diastolic BP (mmHg)	77.5 ± 2.59	80.9 ± 2.51	84.4 ± 4.03	82.0 ± 3.77
Hemoglobin (g/dL)	12.8 ± 1.5	12.3 ± 1.5	11.8 ± 1.4	11.9 ± 1.9
Total protein (g/dL)	7.05 ± 0.52	6.77 ± 0.59	7.00 ± 0.36	6.79 ± 0.59
Serum albumin (g/dL)	4.03 ± 0.42	3.73 ± 0.51	4.15 ± 0.41	3.90 ± 0.42
LDL-cholesterol (mg/dL)	110 ± 43	112 ± 27	112 ± 26	107 ± 3
BUN (mg/dL)	26.8 ± 8.7	36.1 ± 16.1*	31.7 ± 10.2	35.1 ± 15.3
Serum Cr (mg/dL)	1.76 ± 0.74	2.41 ± 1.21	2.48 ± 1.96	2.58 ± 1.27
eGFR (mL/min/1.73m^2^)	34.6 ± 16.1	25.8 ± 10.9	27.1 ± 15.1	24.6 ± 18.6
UP/UCrea (g/gCr)	1.39 ± 1.42	3.06 ± 3.70	0.88 ± 0.75	1.82 ± 1.52
Uric acid (mg/dL)	6.11 ± 1.50	6.02 ± 0.92	6.24 ± 1.25	6.23 ± 0.77

Values are shown as mean ± standard deviation or range

*BP* blood pressure, *eGFR* estimated glomerular filtration rate, *No.* number, *BUN* blood urea nitrogen, *Cr* creatinine, *UP/UCrea* urinary protein/urinary creatinine ratio, *LDL* low-density lipoprotein. *p < 0.05 vs eGFR↑/UP↑ group

### Changes in eGFR after initiation of DAPA according to DAPA-CKD trial criteria

**Fig. 5:**
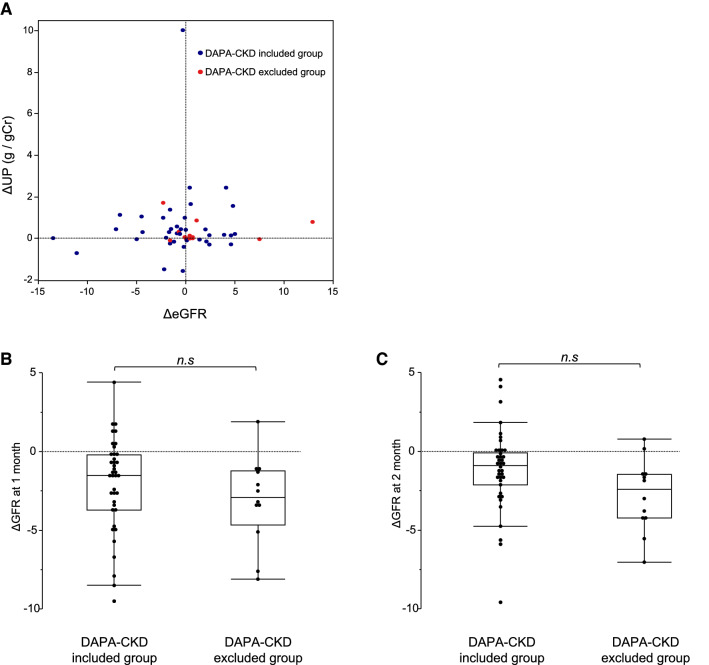
Changes in eGFR after initiation of DAPA in individuals meeting inclusion criteria of DAPA-CKD trial and those meeting exclusion of DAPA-CKD trial. (**A**) Scatter plot showing the current status of changes in eGFR and UP prior to initiation of DAPA between individuals meeting inclusion of DAPA-CKD trial (blue) and those meeting exclusion of DAPA-CKD trial (red). (**B**) The change in eGFR at 1 month and (**C**) 2 months after initiation of DAPA in each group. *CKD* chronic kidney disease, *DAPA* dapagliflozin, *eGFR* estimated glomerular filtration rate, *UP* urinary protein

DAPA-CKD trial included diabetic kidney disease, hypertensive nephropathy, and chronic glomerulonephritis, including IgA nephropathy, membranous nephropathy, focal segmental glomerulosclerosis, and minimal change nephrotic syndrome. By contrast, polycystic kidney disease (PKD), lupus nephritis, and patients receiving immunosuppressive therapy within 6 months prior to enrollment were excluded from DAPA-CKD trial. Thus, we classified the patients into two groups as follows: the individuals meeting inclusion criteria of DAPA-CKD trial and the individuals meeting exclusion criteria of DAPA-CKD trail. There was no difference in the current status of changes in eGFR and UP prior to initiation of DAPA between DAPA-CKD included group and DAPA-CKD excluded group (Fig. 5A). Also, no statistical differences in clinical parameters at baseline, such as age, sex, renal function, anemia, and blood pressure, were observed between the two groups, except for proteinuria (Table 5). ∆eGFR at 1 and 2 months after initiation of DAPA was similar between DAPA-CKD included group and DAPA-CKD excluded group (Fig. 5B, C), suggesting that the initial eGFR dip occurs in individuals excluded from DAPA-CKD trial at the same degree as the individuals included in DAPA-CKD trial.

**Table 5:** Differential characteristics according to the criteria of DAPA-CKD trial

Group group	DAPA-CKD group included	DAPA-CKD excluded
No. of patients	42	9
Age (years)	59.2 ± 14.0	52.1 ± 13.1
Sex (male)	20 (47)	5 (55)
Systolic BP (mmHg)	129.1 ± 14.9	134.4 ± 13.3
Diastolic BP (mmHg)	80.4 ± 10.5	80.4 ± 11.9
Hemoglobin (g/dL)	12.3 ± 1.67	12.3 ± 1.10
Total protein (g/dL)	6.85 ± 0.55	7.13 ± 0.40
Serum albumin (g/dL)	3.85 ± 0.45	4.18 ± 0.47
LDL-cholesterol (mg/dL)	110 ± 33.1	110 ± 32.0
BUN (mg/dL)	32.1 ± 14.1	32.8 ± 9.56
Serum Cr (mg/dL)	2.18 ± 1.30	2.42 ± 0.87
eGFR (mL/min/1.73m^2^)	29.6 ± 15.4	24.6 ± 11.4
UP/UCrea (g/gCr)	2.39 ± 2.75	0.40 ± 0.47***
Uric acid (mg/dL)	5.93 ± 1.06	6.90 ± 1.24

Values are shown as mean ± standard deviation or range

*BP* blood pressure, *eGFR* estimated glomerular filtration rate, *No.* number, *BUN* blood urea nitrogen, *Cr* creatinine, *UP/UCrea* urinary protein/urinary creatinine ratio, *LDL* low-density lipoprotein. ***p < 0.001 vs DAPA-CKD included group

## Discussion

In the present study, we found that oral administration with 10 mg DAPA daily reduced the eGFR from 1 month after starting DAPA, and the reduction sustained until second month in CKD patients without DM. Serum uric acid levels decreased, and hemoglobin levels increased by 2 month DAPA treatment. Baseline eGFR was an independent determinant of the initial eGFR dip in CKD patients without DM. We also identified that the initial eGFR dip was larger in patients currently showing exacerbation of hyperfiltration (increased eGFR and proteinuria) than those currently showing progressive renal dysfunction (decreased eGFR and increased proteinuria). Furthermore, the patients meeting exclusion criteria of DAPA-CKD trial exhibited same degree of the initial eGFR dip as others.

The SGLT2i-induced initial eGFR dip has been reported by several large clinical trials, such as the EMPA-REG outcome [8] and CANVAS program [13] in diabetic population. In the present study, the initial eGFR dip was identified in CKD patients without DM at 1 month after the initiation of DAPA treatment, which is compatible with the results from a pre-specified analysis of the DAPA-CKD trial in patients with IgA nephropathy [14]. The initial eGFR dip in diabetic condition is thought to be induced by a reduction in glomerular pressure, since SGLT2-regulated sodium uptake is increased due to glucosuria, which, in turn, causes tubuloglomerular feedback. Recent evidence has suggested that angiotensin II infusion or upregulation of angiotensinogen increases tubular SGLT2 mRNA levels in a hypertensive rodent model [15]. Thus, SGLT2 may be upregulated not only in diabetic conditions but also in CKD patients without DM possibly due to intrarenal RAS activation. Meanwhile, another hemodynamic mechanism of SGLT2i is also involved in the initial eGFR dip, because the use of RAS inhibitors did not affect eGFR changes and the renal hard outcome in participants with type 2 DM in the J-CKD DB extension study [16]. This should be clarified by further investigations in the future.

Large clinical trials have demonstrated the beneficial effects of SGLT2i on albuminuria in patients with DM [17, 18]. In fact, the DAPA-CKD trial has demonstrated that urinary albumin excretion was dramatically reduced by DAPA treatment within 1 month [19]. In the present study, UP was similar until 2 months of DAPA treatment, while an initial eGFR dip was observed. The difference in clinical characteristics at baseline might have caused this discrepancy. A lesser effect of SGLT2i on urinary albumin excretion has been reported in patients with lower eGFR because of low glucosuria and natriuria [20]. The baseline eGFR in the enrolled patients was found to be lower than that in other trials, which may be linked to insufficient effect of DAPA on proteinuria in the present study. Furthermore, albuminuria is known to be more sensitive marker than proteinuria for monitoring glomerular damage. We enrolled the patients with PKD or lupus nephritis and the patients with glomerulonephritis receiving immunosuppressants, who were excluded from the DAPA-CKD trial. We have compared the effect of DAPA on the initial eGFR dip between the individuals meeting inclusion criteria of DAPA-CKD trial and the individuals meeting exclusion criteria of DAPA-CKD trial. Our analysis demonstrated that there are no significant differences in the initial eGFR dip and proteinuria at 1 and 2 months between them. A longitudinal study needs to be performed to clarify whether DAPA improves proteinuria and the declined eGFR in severe CKD patients without DM.

Hyperuricemia and renal anemia are the major therapeutic targets for CKD patients [21, 22]. In the present study, DAPA significantly reduced serum uric acid levels and increased hemoglobin levels within 1 month, consistent with the class effects reported by a meta-analysis [23]. The mechanisms of these changes induced by DAPA have been comprehensively suggested as follows. The SGLT2i-induced increase in urinary glucose flux may exchange glucose with uric acid at the apical site of proximal tubular cells mainly via GLUT9 isoform 2, leading to further elimination of uric acid into urine [24]. Increased glucosuria is also shown to inhibit reabsorption of uric acid via GLUT9 isoform 2, followed by the decrease in serum uric acid [25]. Previous studies have demonstrated that SGLT2i increases hemoglobin levels in patients with type 2 DM [26, 27]. For instance, hemoglobin level was significantly increased by administration of empagliflozin, accompanied by increased erythropoietin levels and reduced serum ferritin in type 2 DM patients with coronary artery disease in the EMPA-HEART CardioLink-6 randomized clinical trial [28, 29]. Considering that the initial eGFR dip reduces renal tissue oxygen delivery, SGLT2i-caused initial GFR dip may accelerate the production of erythropoietin, leading to the attenuation of renal anemia. Besides, SGLT2i-induced increase in sodium delivery to distal tubules might enhance the expression of hypoxia-inducible factors, which stimulate erythropoiesis. SGLT2i-modulated reduction in oxidative stress and excess energy consumption may affect the synthesis of erythropoietin, as well [28]. Considering that anemia is associated with an increased risk of long-term adverse cardiovascular events and deaths, the attenuation of anemia with SGLT2i can prevent adverse cardiovascular outcomes in CKD population. Furthermore, SGLT2i can presumably inhibit overuse of iron, a risk factor for cardio artery disease, mainly via endothelium dysfunction [30]. Thus, SGLT2i might be a potential to break the crosstalk between heart and kidney and to prevent vicious cycle of cardio-renal-anemia syndrome.

We categorized the patients into four groups based on ∆eGFR and ∆UP before DAPA treatment and then investigated the correlation with the initial eGFR dip. The initial dip of eGFR at 1 month was larger in exacerbation of glomerular hyperfiltration group when compared to that in progressive renal dysfunction group regardless of the baseline eGFR. This finding suggests that SGLT2i is likely to ameliorate glomerular hyperfiltration in CKD patients with larger glomerular hyperfiltration, even though RAS inhibitors are already prescribed. Considering that a large decline in eGFR with DAPA at early stage is linked to prevent long-term renal dysfunction in patients with heart failure [31], a large drop and subsequent recovery in eGFR with DAPA might affect the long-term renal outcome even in CKD population. Further investigation in terms of the correlation between initial dip in eGFR and longitudinal renal outcome will be performed in the future.

## Limitations

The present study is a retrospective single-center study; thus, the causal relationship between the initial eGFR dip and other clinical comorbidities is unknown and there is a confounding by indication bias. It is also undeniable that clinical background of the enrolled patients is slightly different from a representative population of CKD patients without DM. The protective effect of SGLT2i against kidney disease was not evaluated due to short duration of the present study. Therefore, a longitudinal multicenter prospective study must be required to determine whether SGLT2i elicits its reno-protective effect through an initial eGFR dip in CKD patients without DM.

## Conclusion

DAPA daily administration resulted in a significant initial eGFR dip in CKD patients without DM. A higher baseline of eGFR and an increase in both eGFR and proteinuria predict a larger dip in eGFR at 1 month after starting DAPA treatment.
