# Correction to: Effect of dapagliflozin on the initial estimated glomerular filtration rate dip in chronic kidney disease patients without diabetes mellitus

**DOI:** 10.1007/s10157-022-02286-x

**Published:** 2022-11-26

**Authors:** Ryo Shibata, Kensei Taguchi, Yusuke Kaida, Kei Fukami

**Affiliations:** Division of Nephrology, Department of Medicine, Kurume University School of Medicine, 67 Asahi‑Machi, Kurume City, Fukuoka Japan

## Correction to: Clinical and Experimental Nephrology 10.1007/s10157-022-02277-y

In the original publication, the Table 2 has been published incorrectly.

The corrected table is given below:

**Table 2:** Univariate and multiple regression analyses for the determinants of initial eGFR dip in non-DM CKD patients

Variables	Univariate regression			Multiple regression		
	SE	*β*	*p*	SE	*β*	*p*
Age	0.649	0.313	0.025*			
Sex	0.025	0.069	0.631			
Hemoglobin	0.071	− 0.390	0.005**	0.256	− 0.105	0.445
Total protein	0.029	− 0.075	0.639			
Serum albumin	0.023	− 0.201	0.165			
LDL-cholesterol	1.615	− 0.285	0.064			
BUN	0.592	0.425	0.002**	0.040	− 0.072	0.696
Serum Cr	0.055	0.382	0.006**			
eGFR	0.586	− 0.585	< 0.001***	0.038	− 0.614	0.002**
UP/UCrea	0.128	0.056	0.701			
Uric acid	0.128	− 0.016	0.914			

Values are shown as mean ± standard deviation. *R*^2^ = 0.400

*eGFR* estimated glomerular filtration rate, *DM* diabetes mellitus, *CKD* chronic kidney disease, *BUN* blood urea nitrogen, *Cr* creatinine, *UP/UCrea* urinary protein/urinary creatinine ratio, *LDL* low-density lipoprotein

*p < 0.05, **p < 0.01, ***p < 0.001

The original article has been corrected.

